# The Effect of Replacing Wildrye Hay with Mulberry Leaves on the Growth Performance, Blood Metabolites, and Carcass Characteristics of Sheep

**DOI:** 10.3390/ani10112018

**Published:** 2020-11-02

**Authors:** Hua Sun, Yang Luo, Fangfang Zhao, Yaotian Fan, Jingnan Ma, Yaqian Jin, Qirui Hou, Gulzar Ahmed, Hongrong Wang

**Affiliations:** 1 College of Animal Science and Technology, Yangzhou University, Yangzhou 225009, China; sh2665167754@163.com (H.S.); 15380326802@163.com (Y.L.); fangfang3629@sina.com (F.Z.); 15262724618@163.com (Y.F.); ananaqiu@163.com (J.M.); dx120170089@yzu.edu.cn (Y.J.); ahmedgulzar121398@gmail.com (G.A.); 2 The Seri-Cultural Research Institute, Chinese Academy of Agricultural Sciences, Zhenjiang 212018, China; houqr@163.com

**Keywords:** mulberry leaf, Chinese wildrye, digestibility, rumen fermentation, blood parameters, fatty acids

## Abstract

**Simple Summary:**

It is well known that mulberry leaves can be used in animal feed because of their high yield, rich protein content, and palatability. Our study found that partially replacing Chinese wildrye with mulberry leaves could improve meat quality without negatively affecting the growth performance, blood metabolites, and carcass characteristics of sheep. The study suggests that mulberry leaves partially substituted for conventional forage in the diet of sheep may be an effective strategy to alleviate the shortage of high-quality forage sources and to reduce feed costs.

**Abstract:**

The objective of this study was to evaluate the effects of partially substituting for conventional forage, Chinese wildrye (CW), with mulberry leaves (ML) on the growth, digestion, ruminal fermentation, blood metabolites, and meat quality of sheep in a 65-day feedlot study. Thirty-two four-month-old male small-tailed Han sheep (25.15 ± 1.03 kg) were randomly assigned to one of four treatments. The dietary treatments consisted of four proportions of ML (0, 8, 24, and 32%) as a substitute for CW (designated as ML0, ML8, ML24, and ML32, respectively). Rumen digesta and blood samples were collected at day 63 of the trial. Carcass traits were assessed after slaughter at the end of performance period. The results from this study revealed no differences in average daily bodyweight gain (ADG), feed conversion ratio (FCR), and final body weight (FBW) among treatments. The apparent digestibility of dry matter (DM), organic matter (OM), and acid detergent fiber (ADF) was higher in the sheep fed with ML than in those fed CW. The ML24 treatment had a higher digestibility of crude protein (CP) and ether extract (EE). There were no differences (*p* = 0.13) in ruminal pH values among the treatments. However, there was more microbial protein (*p* < 0.01) in ML24 and ML32 treatments than the ML0 treatment. Ruminal concentrations of acetate and butyrate were significantly different among treatments, although no difference in concentrations of total volatile fatty acid were found. Additionally, no differences were detected for serum parameters except blood urea nitrogen (BUN). No differences were observed for carcass weight (*p* = 0.62), dressing percentage (*p* = 0.31) or longissimus dorsi muscle (LM) area (*p* = 0.94) among treatments. However, intramuscular fat was higher in the ML24 treatment than in the ML0 treatment. (*p* < 0.01). There were higher pH values of the 24-h longissimus dorsi in the ML24 treatment than in the ML0 treatment. In addition, the saturated fatty acid (SFA) content was lower (*p* < 0.01) and the monounsaturated fatty acid (MUFA) content higher (*p* < 0.01) in the ML24 treatment than in the ML0 treatment. In conclusion, the partially substitution of mulberry leaves for Chinese wildrye in the diet of sheep had a beneficial influence on the growth performance, blood metabolites and carcass characteristics. The inclusion of 24% (air dry basis) mulberry leaf hay in the ration of sheep is recommended based on these findings.

## 1. Introduction

Chinese wildrye (CW) is a native, cool-season perennial species of gramineae that is mainly distributed in the Eurasian Steppe, including the eastern Inner Mongolian Plateau and the north-eastern China [1]. However, regions such as North and Central China have limited CW forage yield for sheep production. Livestock farms in these regions have to buy higher priced CW due to the long transportation distances involved. Therefore, in the ruminant industry, it is necessary to explore alternative forage sources to prevent forage scarcity and reduce the cost of feed.

The mulberry tree is a multi-purpose species that thrives in a variety of climates from the temperate zone to the tropics. It produces fruit, and its leaves are mainly used for sericulture, traditional herbs, and animal feeds [2]. The cultivated area of mulberry is estimated to be more than one million hectares, and the biological yield of fresh mulberry leaves is about 25–30 tons/hectare/year in China [3]. In recent years, mulberry leaves and its residuals were potentially applied as ruminant forage. One alternative is to revalue and use mulberry waste in the form of dried pellets that can be used in animal feed, especially as the properties of high yield, rich protein content, good palatability and high digestibility in ruminant rations support growth and lactation [4,5]. Mulberry leaf silage is also used in the diet to improve meat quality and reduce feed costs [6]. In addition, it has been found that mulberry leaves contain a variety of active substances, including polysaccharides, flavonoids, and polyphenols, which can reduce blood glucose and triglycerides, and also have the function of antioxidation and regulation of lipid metabolism [7,8,9,10]. Mulberry leaves and their residuals can be used to supplement the forage of ruminants when a high-quality forage source is limited [11]. Several studies have shown that ML can increase feed utilization and improve rumen fermentation in beef cattle [6,12]. Huyen et al. reported that mulberry leaf pellet supplementation can lead to increased nutrient digestibility [13]. Furthermore, it was reported that a mulberry leaf powder supplement can promote the development and metabolic properties of rumen epithelium [14]. Therefore, replacement of ML may important and meaningful. However, there is little published information on the effects of ML as a CW replacement.

The use of local alternative feeds can offer economic advantages by reducing feeding costs and mitigating the adverse socio-environmental impacts that would otherwise arise from the disposal of agri-industrial by-products. Our hypothesis was that ML may replace CW in the diets of small-tailed Han sheep without having a negative effect. The objective of this study was to investigate the effect of partially replacing Chinese wildrye with mulberry leaves in the diet on the growth performance, blood metabolites, and carcass characteristics of small-tailed Han sheep.

## 2. Materials and Methods

This study was performed in accordance with the approved Regulations for the Administration of Affairs concerning Experimental Animals [15]. The experimental procedures were approved by the Animal Welfare and Ethics Committee of Yangzhou University (YZUDWLL-201701-001).

### 2.1. Animals, Diets, and Experimental Procedure

The feeding experiment was conducted at the farm of Yangzhou University (Yangzhou, Jiangsu, China). The ML was sourced from the sericulture Research Institute of the Chinese Academy of Agricultural Sciences, which was carefully dried by air and kept for the feeding trial. The CW was acquired from Northeast China through line haul.

Thirty-two small-tailed Han sheep (four-month-old rams, BW = 25.15 ± 1.03 kg) were used for the feeding trial and were assigned to one of four dietary treatments in a randomized complete block design experiment. Animals were raised in individual pens and offered a total mixed ration (TMR) composed of 60% concentrate and 40% forage, including four proportions of replacement of CW with ML (0, 8%, 24%, and 32%). The 16% proportions of mulberry leaves in the dietary was not used in this trial because it shows a negative effect in rumen fermentation in vitro in our companion paper. Experimental diets met the requirements of all animals for growth and BW gain according to the NRC [16]. Dietary ingredients and nutrient compositions used in the trial are shown in Table 1. The experimental period lasted 65 days with the first 10 days for adaptation and the subsequent 55 days for measurements. Feed was offered twice daily at approximately 700 h and 1700 h, and animals had ad libitum access to water and a trace mineral salt block. The amount of feed was adjusted every 3 to 4 d, permitting 5 to 10% residues. Feed intake and refused feed were treated as daily repeated measures for 65 d for growth performance, and the body weight (BW) of each sheep was recorded weekly in the early morning before feeding, and the average daily gain (ADG) was calculated as the regression of BW measured over time. The feed conversion ratio (FCR) was calculated as DMI (g/d) divided by ADG (g/d).

### 2.2. Sampling

Feed offered and refused was recorded daily, and samples of diets and orts were collected daily and pooled by treatment. The samples were dried at 65 °C for 48 h in an oven, ground through a 1 mm screen using a Wiley mill (A. H. Thomas Co., Philadelphia, PA, USA), and kept at −20 °C until analysis for DM, OM, CP, ether extract, NDF, and ADF. During the last week of the experiment, four sheep from each treatment with similar DMI and BW were selected for a 4-d digestibility test. Sixteen fecal-collection containers were prepared and were responsible for collecting fecal samples of individual sheep. After every 24 h, the total fecal matter collected was weighed, mixed, and recorded. About 2% of the mixed feces was sampled, oven dried at 65 °C for 48 h, ground through a 1 mm screen (Wiley mill), and stored at −20 °C for later analysis of DM, OM, CP, NDF, and ADF.

On day 63 of the experimental period, blood was obtained via the jugular vein into 10 mL evacuated serum tubes before the morning feeding. Serum samples were obtained after the tubes were centrifuged at 3000× *g* at 4 °C for 20 min. Next, the serum was separated into three aliquots and was kept at −20 °C for biochemical indicators.

Approximately 100 mL of ruminal sample, consisting of a mixture of liquids and solids, was obtained from a stomach tube connected to a vacuum pump through the esophagus at 2 h after morning feeding on day 63. The pH value was immediately determined using a portable pH meter (HJ-90B, Aerospace Computer Company, Beijing, China). Then, 0.25 mL of metaphosphoric acid (25 g/100 mL) was added to four aliquots of 1 mL rumen fluid, which were centrifuged at 15,000× *g* at 4 °C for 15 min to determine the VFA and NH_3_–N concentrations.

At the end of the feeding trial, animals were slaughtered according to block across treatments four animals from each treatment). Sheep final BW (FBW) was recorded prior to slaughter to determine dressing percentage. Carcass weight was obtained on the day of slaughter. Carcasses were subsequently stored at 4 °C for 48 h. The left half carcass was transported to the meat quality laboratory. Carcasses were cut between the 12th and 13th ribs for assessment of carcass quality attributes. Carcass quality attributes included 12th-rib fat thickness, longissimus dorsi muscle (LM) area, intramuscular fat, and ultimate pH values. Carcass color was assessed by measuring L*, a*, and b* color values using a portable Minolta chromameter (Minolta Chroma Meter CR-400 colorimeter, Minolta Corp., Osaka, Japan) on the cut lean surface and carcass external fat along the lateral side of the carcass. Color readings were recorded in the L* (0 = black, 100 = white), a* (negative values = green, positive values = red), and b* (negative values = blue, positive values = yellow) color space (CIELAB); large pieces of connective tissue and intramuscular fat were avoided. Color saturation was calculated as described by the operational manual [17]. The pH of the LM was measured by making a scalpel incision and inserting a glass electrode (Model EC2010-11, Amargruss Electrodes Ltd., Castlebar, Co. Mayo, Ireland) attached to a portable pH meter (Model no. 250 A, Orion Research Inc., Boston, MA, USA) 2.5 cm into the muscle. One steak (all 2.5 cm thick) was cut for immediate measurement of drip-loss according to the procedure of Honikel [18], one steak was cut, vacuum packed and stored at −18 °C for subsequent Warner–Bratzler shear force (WBSF) measurement according to the procedure of Shackelford, Koohmarie, and Savell [19]. Briefly, frozen vacuum-packed steaks were thawed in a circulating water bath at 10–15 °C and allowed to equilibrate to ambient temperature. Steaks were cooked in retortable vacuum pack bags to an internal temperature of 70 °C, by immersing in a water bath (Model HH-6, LICHEN Instruments Ltd.) at 80 °C. Five cores (1.25 cm diameter) were cut fromthe steaks parallel to the direction of the muscle fibres and sheared using an Instron Universal testing machine equipped with a triangular Warner–Bratzler shearing device. Cooking loss, expressed as a percentage of weight loss, was calculated from the ratio between the initial weight (before cooking) and the final weight (after cooking).

For fatty acid analysis, intramuscular fat was extracted from blended LM using the modified version of Folch, Lees, and Stanley [20] described by Noci et al. [21]. Fatty acids were quantified as their fatty acid methyl esters as described by French et al. [22]. Individual fatty acids were identified by retention time with reference to fatty acid standards.

### 2.3. Analytical Procedures

The DM and ash of feed samples were determined according to methods 934.01 and 924.05, respectively, of AOAC (1990) [23]. The OM content was calculated as the total percentage (100%) minus the ash content. The CP was analyzed by the Kjeldahl method (AOAC, 1990; method 990.03) using a Kjeldahl nitrogen determination apparatus (Kjeltec 2100, Foss, Hillerod, Denmark). The ether extract was determined in accordance with AOAC (1990) method 920.39 using an AnkomXT15 Extractor (Ankom Technology, Fairport, NY, USA). An Ankom fiber analyzer (Ankom Technology) was used to examine ADF and NDF according to the method described by Van Soest et al. (1991) [24], and α-amylase and sodium sulfite were used for the NDF procedure.

The biochemical indicators of serum were analyzed using an automated biochemistry analyser (Hitachi 7020; Hitachi Co., Tokyo, Japan). The concentrations of glucose, triglyceride, total protein and urea nitrogen were measured using glucose oxidase, glycerophosphate oxidase, biuret and urease, respectively, applying the commercial test kits according to the manufacturer’s instructions (Jiuqiang Bio-Technique Co., Beijing, China). The total cholesterol, albumin, high-density lipoprotein cholesterol (HDL-C), and low-density lipoprotein cholesterol (LDL-C) concentrations in serum were analyzed using an enzymatic method, a bromocresol green method, and a direct measurement method, respectively.

Ruminal NH_3_-N concentration was measured according to Bremner et al. [25] using a spectrophotometer (UV-1700, Shimadzu Corporation, Kyoto, Japan). Microbial crude protein (MCP) was measured according to Wang et al. [26], The concentration of ruminal VFA was determined by GC as described by Huo et al. [27] Briefly, samples passed through a 0.45 um filter, and were transferred into a capillary column GC (GC-14B, Shimadzu; film thickness of the capillary column, 30 m × 0.32 mm × 0.25 mm; column temperature, 110 °C; injector temperature, 180 °C; and detector temperature, 180 °C).

### 2.4. Statistical Analyses

Data were subjected to analysis of variance as a completely randomized design using the general linear model (GLM) procedure of SAS (SAS Inst., Inc., Cary, NC, USA). Sheep were used as the experimental units. Means were separated by Duncan’s multiple range tests when a significant treatment effect was observed. Linear and quadratic regression analyses were done using the CONTRAST statement of SAS based on the dietary mulberry leaf proportion. Results were considered statistically significant at *p* < 0.05.

## 3. Result

### 3.1. Performance, Intake, and Digestibility

The results of dry matter intake (DMI), growth performance, and apparent digestibility are listed in Table 2 and Table 3. There were no differences among treatments in terms of average daily gain (ADG), feed conversion ratio (FCR), and final body weight (BW). However, DMI and average daily feed intake (ADFI) were numerically higher in the ML24 treatment than in the ML0 treatment. The apparent digestibility of DM, OM, and ADF was higher in the ML treatment than in the CW treatment. Additionally, sheep from the ML24 group had a significantly higher apparent digestibility of CP, NDF, and EE (*p* < 0.01) than sheep from the ML0 group.

### 3.2. Rumen Fermentation

The parameters of the rumen fermentation characteristics are presented in Table 4. The pH values of rumen liquid were similar among treatments. However, the MCP concentration was higher in the ML24 and ML32 treatments than in the ML0 treatment. There were no differences in the concentrations of NH_3_-N, total volatile fatty acids (TVFA), propionate, or in the acetate/propionate ratio among treatments. The concentration of acetate was higher (*p* = 0.04) in the ML0 treatment than in the ML24 and ML32 treatments. However, the ML0 treatment had a lower butyrate concentration than other treatments.

### 3.3. Blood Metabolites

The effects of the dietary treatments on serum biochemical indicators are presented in Table 5. The ML0 treatment had a higher level of urea N among treatments. The dietary treatments exhibited no differences in serum glucose, triglycerides, cholesterol, total protein, albumin, HDL-C, and LDL-C concentrations.

### 3.4. Carcass Characteristics, Meat Quality, and Fatty Acids

The carcass characteristics of the four experimental treatments are shown in Table 6. No difference (*p* > 0.05) were observed in FBW, carcass weight, dressing percentage, and longissimus dorsi muscle area (LMA) among treatments. However, the 12th-rib fat thickness was higher in the ML32 treatment than in the ML0 treatment, and the IMF percentage was also greater in ML24 and ML32 treatment than ML0 treatment. Carcass color and other meat quality characteristics are shown in Table 7. There were no differences in LM a* values and b* values among the treatments. However, LM from the CW dietary treatment had higher L* values than that from the ML dietary treatment. There were no differences in pH values of the 24-h LM among the treatments; pH values were within the normal pH range. Additionally, LM from ML24 had a lower drip loss at 24 h than LM from the ML0 treatment.

Muscle fatty acid composition data are summarized in Table 8. The proportion of C15:1, C18:In9c, C18:3n3, MUFA, and the PUFA: SFA ratio in LM were higher in the ML24 treatment than in the ML0 treatment, and the proportion of C16:0 and SFA were lower in the ML24 treatment than in other treatments.

## 4. Discussion

Feed intake can be affected by both physiological factors and external factors, including dietary chemical composition, environmental conditions, and animal learning abilities [28,29]. Regarding chemical components, DMI generally decreases with increasing NDF concentration [30]. In the present study, the DMI was numerically higher in treatments that included ML in the diet. This may be due to the low NDF and high crude protein contents in mulberry leaves, making this forage a palatable feed for ruminants. [4] Steen et al. [31] reported that digestibility was a crucial factor in regulating forage intake. This finding is consistent with the present study, namely, that the ML24 treatment had higher apparent digestibility of DM. Polyphenols are substrates for several enzymes, including hydrolyzing and conjugating enzymes, and they are located in the small intestine and colon [32]. In this study, the higher DM and OM digestibility in the ML treatment might be explained by plant polyphenols in mulberry leaves. Niu [33] and Uribe [34] observed that cattle fed silage mulberry leaves could have a higher abundance of ruminal bacteria and higher reproduction of fiber-degrading bacteria, which could be a reason for the higher digestibility of NDF and NDF in the ML treatment. Additionally, the *Ruminococcus flavefaciens* and *Butyrivibrio fibrisolvens*, two of the main ruminal cellulolytic species, degrade cellulose and hemicellulose, exposing plant proteins [35,36,37]. The proteolytic activity of *B. fibrisolvens* [38] might have also directly contributed to the increased CP degradation. Moreover, *B. fibrisolvens* is a major ruminal lipolytic bacterial species, and it participates in the biohydrogenation of feed lipids [39], which probably explains the higher digestibility of the ether extract.

Ruminal pH is an important indicator of the rumen microbial ecosystem. Lower ruminal pH is a limiting factor to the establishment of a balanced microbial population and has a negative effect on fiber digestion via reduced microbial attachment [40,41]. Our results showed that replacing CW with ML did not change the average ruminal fluid pH, but that values were in the optimal range for microbial digestion of fiber and protein [42]. Ruminal microbial protein and VFA supply much of the protein and energy needs of ruminants. Furthermore, rumen microbial proteins are mainly derived from rumen microbe. Our study showed that higher concentrations of MCP in the ML24 and ML32 treatments, which may be due to an enhanced abundance of ruminal bacteria [33]. Bach et al. [43] found that the concentration of acetate was increased when fiber intake increased. Therefore, the higher acetate concentration could be attributed to the high fiber content in the ML0 diet. The total VFA concentration did not differ among treatments, which might be the result of the same ratio of forage to concentrate of diets [44], or might be caused by the fact that the output of total VFA was equivalent to the amount of absorption in the rumen among treatments [45]. In the present research, the observed concentrations of propionate and the acetate to propionate ratio were not significantly different, probably because of the similar ruminal pH values caused by dietary-associated shifts [46].

For ruminants, the primary substrate of hepatic gluconeogenesis is propionate from rumen fermentation [47,48]. In the present study, the concentrations of plasma glucose and triglyceride were similar among treatments, potentially due to the similar ruminal propionate concentrations. Changes in diets did not affect the concentrations of cholesterol, HDL-C, and LDL-C in serum, which indicated no impairment of the heart and liver of the sheep [12]. The similar serum cholesterol concentration is likely attributable to the similar concentrations of HDL-C and LDL-C [49] because blood cholesterol is related to the concentration of HDL-C and LDL-C in cattle [50]. The similarities in serum albumin and total protein concentrations among the dietary treatments can probably be explained by the similar flow of microbial protein to the intestine [51] and the amount of AA available for absorption [52]. Plasma urea nitrogen is the end product of proteolysis and amino acid metabolism; its concentration is related to crude protein levels. The ammonia produced by the degradation of crude protein in the rumen is used to synthesize microbial protein, and the rest of the ammonia is absorbed into the blood through the rumen wall and enters the liver to synthesize urea. In the current study, the ML0 treatment had a higher plasma urea concentration than the other treatments, which may be attributed to the lower microbial protein concentration of that treatment.

There are many individual sensory attributes that influence the acceptability of meat (e.g, juiciness, colour, greasiness, etc.). However, an overall appraisal provides an idea of whether or not the consumers like the meat. In the present study, the effect of including ML in sheep feed on the overall acceptability of sheep meat was insignificant in terms of FBW, carcass weight, dressing percentage, and LM area. Similar carcass characteristics revealed that there were no adverse effects of including ML in the diet. Additionally, switching from a conventional diet to a partially substituted diet can change the amount of fat deposited in sheep. The 12th-rib fat thickness was higher in the ML24 treatment, probably due to the higher butyrate concentrations. This finding may be consistent with the fact that butyrate is the precursor substance of fat synthesis [45].

With regard to the meat colour, replacing CW with ML in the present study had no effect on the a* values and b* values of LM. However, the ML treatments had lower L* values of LM. It is reported that lambs fed a diet containing tannins had a lighter color (higher L*) of LM with decreased blood hemoglobin concentrations than those fed a non-tannins diet [53]. The lower L* values could be attributed to the low tannins content of the ML diet [54]. In the present study, no significant differences (*p* > 0.05) were observed in meat pH values after 24 h for the four treatments. The use of ML in the sheep feed did not seem to affect the expected pH values of the sheep meat, so alterations to the properties of the meat during maturation are not expected. Interestingly, current study found that the IMF percentage was also greater in the ML24 and the ML32 treatments than ML0 treatment. This finding may be related to the fact that mulberry leaf flavonoids can increase insulin levels in animals [8]. Insulin is a metabolic hormone that can promote fat production and promote IMF cells to preferentially use a carbohydrate carbon framework to synthesize FA [55].

Consumers are increasingly interested in the lipid content of edible meat due to its relationship with human health. Meat is regarded as a major source of fatty acids for the human diet. While saturated fatty acid (SFA) intake is a risk factor for cardiovascular disease, maximizing the PUFA proportion, and in particular the n−3 PUFA proportion of lipids, would enhance the nutritive value of meat by reducing the incidence of hypertension, cardiovascular disease, and arthritis [56,57]. In this study, significant differences were observed in the PUFA/SFA ratio. Moreover, the ML24 dietary treatment had higher proportions of mono unsaturated fatty acid (MUFA) and the ratio of PUFA to SFA compared with other treatments. This is consistent with the finding of Jeon et al. [58], who reported that steer fed a mulberry silage supplementation diet had a higher proportion of PUFA than those fed non-mulberry silage supplementation diets. The diet is the main factor that affect carcass and intramuscular fatty acid composition [59]. Modifying diet energy density and increasing the supply of unsaturated fatty acids affect the muscle fatty acid composition [60]. Although the four treatments had similar metabolizable energy (ME), higher proportions of MUFA and the PUFA: SFA ratio was measured in the ML24 treatment than in the ML0 treatment, which could be considered preferable for human health.

## 5. Conclusions

Replacing around 24–32% of conventional forage with mulberry leaves in the diet of sheep may be recommended given that there are unlikely to be any significant changes in the physical and chemical properties of the meat. This may promote the purchase intention of consumers due to the improved color and intramuscular fat of the meat. Results from this study indicate that partially replacing forage with mulberry leaves may improve meat quality without negatively affecting the growth performance, blood metabolites and carcass characteristics of sheep. The use of mulberry leaves at 24% of forage as the optimal substitution rate in diet may be proposed as a strategy to improve sheep production.

## Figures and Tables

**Table 1:** Dietary composition and nutrient level in the experimental treatments of sheep.

Item	Treatments			
	^1^ ML0	ML8	ML24	ML32
Ingredients				
Mulberry leaf hay	0.00	8.00	24.00	32.00
Chinese wildrye hay	40.00	32.00	16.00	8.00
Corn	40.50	38.50	40.50	43.80
Wheat bran	0.00	4.50	6.90	10.80
Soybean meal	18.00	15.50	11.10	3.90
NaCl	0.50	0.50	0.50	0.50
Premix ^2^	1.00	1.00	1.00	1.00
Total	100.00	100.00	100.00	100.00
Nutrient levels ^3^				
ME (MJ/Kg)	10.08	10.07	10.16	10.16
CP %	13.32	13.94	14.90	13.77
EE %	4.16	4.38	4.78	5.07
NDF %	35.36	33.32	27.35	24.85
ADF %	18.62	17.05	13.30	11.35
Ca %	0.13	0.24	0.45	0.54
P %	0.24	0.27	0.27	0.28
Chemical composition	Mulberry leaves		Chinese wildrye	
ME (MJ/Kg)	9.04			8.18
CP %	20.30			5.50
EE %	34.00			74.00
NDF %	16.00			39.00
ADF %	8.00			6.00
Ca %	1.54			0.16
P %	0.098			0.055

^1^ ML0, diet based on cereal concentrates with Chinese wildrye hay; ML8, diet based on concentrate, including 8% replacement of Chinese wildrye hay by Mulberry leaf hay; ML24, diet based on concentrate, including 24% replacement of Chinese wildrye hay by Mulberry leaf hay; ML32, diet based on concentrate, including 32% replacement of Chinese wildrye hay by Mulberry leaf hay. ^2^ One kg of premix contained the following: VA150~200KIU, VD_3_45~80KDU, VE ≥ 200 mg, niacin ≥ 400 mg, Zn 1.0–2.0 g, Cu 0.1–0.2 g, Mn 1.0–3.0 g, Fe 0.8–1.6 g, Se 4.0–8.0 mg, Ca 10.0–20.0%, *p* ≥ 1.8%, NaCl5.0~10.0%. ^3^ All values are analyzed values except metabolizable energy, Ca, and P.

**Table 2:** Effects of different dietary mulberry leaf hay proportion treatments on the dry matter intake and growth performance of sheep.

Item	Treatments				SEM ^1^	*p*-Value	
	ML0	ML8	ML24	ML32		Linear	Quadratic
DMI (g)	1534.5	1604.5	1673.2	1600.7	59.573	0.67	0.61
IBW (kg)	24.93	25.30	24.91	25.46	0.348	0.71	0.91
FBW (kg)	38.90	39.18	39.21	40.03	0.335	0.28	0.70
ADFI	1215.79	1230.80	1260.56	1239.53	7.157	0.11	0.20
ADG (g)	254.4	252.21	260.05	264.71	0.007	0.53	0.81
FCR	4.88	4.98	4.90	4.82	0.134	0.85	0.75

^1^ SEM = standard error of means; DMI = dry matter intake; IBW = initial body weight; FBW = final body weight; ADG = average daily gain; ADFI = average daily feed intake; FCR = feed conversion ratio.

**Table 3:** Effects of inclusive ML treatments on the apparent digestibility of dietary nutrients in sheep.

Item	Treatments				SEM ^1^	*p*-Value	
	ML0	ML8	ML24	ML32		Linear	Quadratic
DM	58.25 ^b^	67.81 ^a^	71.87 ^a^	67.51 ^a^	0.017	<0.01	<0.01
OM	63.06 ^c^	71.79 ^b^	76.76 ^a^	73.76 ^ab^	0.016	<0.01	<0.01
CP	61.26 ^b^	70.45 ^a^	73.14 ^a^	60.72 ^b^	0.017	0.810	<0.01
NDF	55.52 ^c^	57.39 ^c^	66.24 ^a^	63.36 ^b^	0.013	<0.01	0.01
ADF	53.30 ^c^	56.99 ^b^	69.20 ^a^	58.48 ^b^	0.018	<0.01	<0.01
EE	65.81 ^b^	58.49 ^c^	72.25 ^a^	70.18 ^a^	0.017	<0.01	0.085

^1^ SEM = standard error of means; DM = dry matter; OM = organic matter; CP = crude protein; NDF = neutral detergent fiber; ADF = acid detergent fiber; EE = ether extract. ^a–c^ Means followed by different superscripts are significantly different (*p* < 0.05).

**Table 4:** Effects of inclusive ML treatments on the rumen fermentation of sheep.

Item	Treatments				SEM ^1^	*p*-Value	
	ML0	ML8	ML24	ML32		Linear	Quadratic
pH	7.12	7.06	6.87	7.08	0.041	0.39	0.09
NH_3_-N (mg/dL)	12.92	11.83	16.49	14.32	0.722	0.15	0.69
MCP (mg/dL)	6.24 ^b^	5.98 ^b^	10.48 ^a^	11.48 ^a^	0.604	<0.01	0.37
TVFA (mM)	96.60	102.51	111.09	108.69	2.661	0.06	0.43
Acetate (mM)	70.95 ^a^	68.06 ^ab^	65.32 ^b^	65.75 ^b^	0.796	0.01	0.25
Propionate (mM)	18.78	19.32	19.58	20.80	0.688	0.34	0.82
Butyrate(mM)	10.27 ^c^	12.62 ^b^	15.10 ^a^	13.45 ^ab^	0.487	<0.01	<0.01
Acetate/Propionate	3.84	3.53	3.59	3.22	0.136	0.16	0.91

^1^ SEM = standard error of means; MCP = microbial protein; TVFA = acetate + propionate + butyrate + valerate + isobutyrate + isovalerate. ^a–c^ Means followed by different superscripts are significantly different (*p* < 0.05).

**Table 5:** Effects of inclusive ML treatments on blood metabolites of sheep.

Item	Treatments				SEM ^1^	*p*-Value	
	ML0	ML8	ML24	ML32		Linear	Quadratic
TP (g/L)	56.50	56.37	57.75	55.38	0.458	0.63	0.23
ALB (g/L)	23.48	24.10	23.77	23.22	0.246	0.62	0.26
BUN (μ mol/L)	8.88 ^a^	6.37 ^b^	6.87 ^ab^	5.87 ^b^	0.436	0.02	0.34
GLU (μ mol/L)	3.46	3.53	3.53	3.49	0.055	0.89	0.67
CHO (μ mol/L)	1.60	1.57	1.39	1.41	0.043	0.11	0.39
TG (μ mol/L)	0.32	0.36	0.46	0.41	0.028	0.16	0.43
HDL (μ mol/L)	0.77	0.68	0.69	0.70	0.021	0.35	0.24
LDL (U/L)	0.54	0.46	0.44	0.44	0.019	0.06	0.29

^1^ SEM = standard error of means; TP = total protein; ALB = albumin; BUN = blood urea nitrogen; GLU = glucose; CHO = total cholesterol; TG = triglyceride; HDL = high-density lipoprotein; LDL = low-density lipoprotein. ^a,b^ Means followed by different superscripts are significantly different (*p* < 0.05).

**Table 6:** Effects of inclusive ML treatments on carcass characteristics of sheep.

Item	Treatments				SEM ^1^	*p*-Value	
	ML0	ML8	ML24	ML32		Linear Quadratic	
Carcass weight (kg)	17.03	17.15	17.83	17.15	0.222	0.62	0.40
Dressing percentage %	44.41	44.80	46.62	44.01	0.005	0.90	0.16
LM area (cm^2^)	23.23	23.86	23.21	23.95	0.499	0.77	0.96
Back fat thickness (mm)	3.65 ^bc^	3.18 ^c^	4.27 ^ab^	4.52 ^a^	0.017	<0.01	0.15
IMF content %	5.72 ^b^	5.52 ^b^	7.85 ^a^	8.00 ^a^	0.004	<0.01	0.69

^1^ SEM = standard error of means; LM = longissimus dorsi muscle; IMF = intramuscular fat. ^a–c^ Means followed by different superscripts are significantly different (*p* < 0.05).

**Table 7:** Effects of inclusive ML treatments on meat quality of sheep.

Item	Treatments				SEM ^1^	*p*-Value	
	ML0	ML8	ML24	ML32		Linear	Quadratic
L*	40.36 ^a^	34.95 ^b^	35.68 ^b^	33.72 ^b^	0.797	<0.01	0.12
a*	13.45	14.76	16.39	14.96	0.475	0.14	0.14
b*	4.91	4.86	4.65	4.40	0.115	0.12	0.67
pH_24 h_	5.65 ^b^	5.65 ^b^	5.68 ^a^	5.66 ^b^	0.004	0.01	0.02
Drip loss %	13.72 ^b^	21.26 ^a^	9.51 ^c^	11.29 ^c^	1.190	<0.01	<0.01
Cook loss %	29.17	21.54	32.09	27.85	1.066	0.11	0.07
Shear force N	37.12	37.21	31.30	38.94	1.149	0.96	0.08

^1^ SEM = standard error of means; color measurements: L* = lightness, black (0) to white (100); positive a* = red; negative a* = green; positive b* = yellow; negative b* = blue. ^a–c^ Means followed by different superscripts are significantly different (*p* < 0.05).

**Table 8:** Effects of inclusive ML treatments on fatty acid composition in longissimus dorsi muscle of sheep (%).

Item	Treatments				SEM ^1^	*p-*Value	
	ML0	ML8	ML24	ML32		Linear	Quadratic
C 10:0	0.08	0.09	0.06	0.06	0.006	0.06	0.87
C 12:0	0.11	0.08	0.12	0.13	0.009	0.09	0.23
C 14:0	1.63	1.61	2.15	2.94	0.368	0.21	0.61
C 14:1	0.10	0.11	0.10	0.16	0.010	0.07	0.21
C 15:0	0.17	0.11	0.16	0.11	0.014	0.37	0.97
C 15:1	15.10 ^c^	17.71 ^b^	21.99 ^a^	18.09 ^b^	0.706	<0.01	<0.01
C 16:0	33.23 ^a^	29.54 ^a^	15.10 ^b^	22.07 ^b^	2.099	<0.01	<0.05
C 16:1	0.65 ^b^	9.57 ^b^	1.34 ^a^	1.47 ^a^	0.133	<0.01	0.58
C 17:0	0.52	0.44	0.58	0.52	0.026	0.55	0.79
C 17:1	10.25 ^b^	11.86 ^ab^	13.33 ^a^	11.50 ^ab^	0.412	0.11	0.02
C 18:0	1.11	1.01	0.74	0.87	0.067	0.11	0.40
C 18: ln9t	30.35	30.62	37.84	34.14	1.364	0.12	0.44
C 18: ln9c	0.34 ^c^	0.40^c^	0.77 ^b^	1.42 ^a^	0.120	<0.01	0.02
C 18:2n6t	0.24	0.26	0.24	0.21	0.023	0.58	0.58
C 18:2n6c	2.44	2.28	3.09	3.28	0.259	0.18	0.75
C 20:0	0.10	0.08	0.04	0.06	0.008	0.03	0.17
C 18:3n6	0.09	0.08	0.05	0.08	0.006	0.19	0.27
C 20: ln9	0.25	0.25	0.13	0.22	0.017	0.10	0.07
C 18:3n3	0.11 ^b^	0.14 ^b^	0.23 ^a^	0.09 ^b^	0.015	0.73	<0.01
C 20:2	0.08	0.09	0.07	0.06	0.007	0.24	0.32
C 22:0	0.06	0.05	0.04	0.06	0.005	0.96	0.26
C 20:3n6	0.03	0.03	0.02	0.04	0.003	0.38	0.46
C 20:3n3	2.27	2.13	1.45	1.92	0.136	0.14	0.24
C 20:4n6	0.70 ^a^	0.55 ^ab^	0.35 ^c^	0.49 ^bc^	0.042	<0.01	0.03
SFA	36.99 ^a^	33.01 ^ab^	18.99 ^c^	26.83 ^b^	2.046	<0.01	0.03
MUFA	57.05 ^c^	61.53 ^bc^	75.49 ^a^	67.01 ^b^	2.117	<0.01	0.03
PUFA	5.96	5.57	5.51	6.16	0.234	0.82	0.32
PUFA/SFA	0.16 ^c^	0.17 ^bc^	0.29^a^	0.23 ^b^	0.016	<0.01	0.08
n-6 PUFA	3.50	3.21	3.76	4.09	0.246	0.33	0.56
n-3 PUFA	2.39	2.27	1.68	2.01	0.128	0.14	0.37
n-6 PUFA/n-3 PUFA	1.57	1.55	2.27	2.10	0.189	0.20	0.86

^1^ SEM = standard error of means; SFA (saturated fatty acid) = C10:0 + C12:0 + C14:0 + C15:0 + C16:0 + C17:0 + C18:0 + C20:0; MUFA (monounsaturated fatty acid) = C14:1 + C15:1 + C16:1 + C17:1 + C18:ln9t + C18:ln9c + C20:ln9; PUFA(polyunsaturated fatty acid) = C18:2n6t + C18:2n6c + C18:3n6 + C18:3n3 + C20:2 + C20:3n6 + C20:3n3 + C20:4n6; n-6PUFA = C18:2n6t + C18:2n6c + C18:3n6 + C20:3n6 + C20:4n6; n-3PUFA = C18:3n3 + C20:3n3; ^a–c^ Means followed by different superscripts are significantly different (*p* < 0.05).
